# A Systematic Review of the Molecular and Cellular Alterations Induced by Cannabis That May Serve as Risk Factors for Bipolar Disorder

**DOI:** 10.1093/ijnp/pyae002

**Published:** 2024-01-04

**Authors:** Alejandra Delgado-Sequera, Clara Garcia-Mompo, Ana Gonzalez-Pinto, Maria Hidalgo-Figueroa, Esther Berrocoso

**Affiliations:** Biomedical Research and Innovation Institute of Cadiz (INiBICA), Research Unit, Puerta del Mar University Hospital, Cádiz, Spain; Neuropsychopharmacology and Psychobiology Research Group, Universidad de Cádiz, Cádiz, Spain; Department of Medicine, School of Medical Sciences, Universitat Jaume I, Castellón de la Plana, Spain; Centro de Investigación Biomédica en Red de Salud Mental (CIBERSAM), ISCIII, Madrid, Spain; Department of Psychiatry, Hospital Universitario de Alava, BIOARABA, UPV/EHU, CIBERSAM, Vitoria-Gasteiz, Spain; Biomedical Research and Innovation Institute of Cadiz (INiBICA), Research Unit, Puerta del Mar University Hospital, Cádiz, Spain; Neuropsychopharmacology and Psychobiology Research Group, Universidad de Cádiz, Cádiz, Spain; Department of Psychology, Universidad de Cádiz, Puerto Real (Cádiz), Spain; Centro de Investigación Biomédica en Red de Salud Mental (CIBERSAM), ISCIII, Madrid, Spain; Biomedical Research and Innovation Institute of Cadiz (INiBICA), Research Unit, Puerta del Mar University Hospital, Cádiz, Spain; Neuropsychopharmacology and Psychobiology Research Group, Universidad de Cádiz, Cádiz, Spain; Department of Neuroscience, Universidad de Cádiz, Cádiz, Spain; Centro de Investigación Biomédica en Red de Salud Mental (CIBERSAM), ISCIII, Madrid, Spain

**Keywords:** Cannabis, bipolar disorder, risk factor, biomarker

## Abstract

**Background:**

Cannabis use is a risk factor of psychiatric illness, such as bipolar disorder type-I (BDI). Indeed, cannabis use strongly influences the onset and clinical course of BDI, although the biological mechanisms underlying this interaction remain unknown. Therefore, we have reviewed the biological mechanisms affected by cannabis use that may trigger BD.

**Methods:**

A systematic review was carried out of articles in which gene expression was studied in cannabis users or human-derived cells exposed to tetrahydrocannabinol (THC) or cannabidiol (CBD). A second systematic review was then performed to identify articles in which gene expression was studied in BDI samples, highlighting those that described alterations to the same molecular and cellular mechanisms affected by cannabis/THC/CBD.

**Results:**

The initial search identified 82 studies on cannabis and 962 on BDI. After removing duplicates and applying the inclusion/exclusion criteria, 9 studies into cannabis and 228 on BDI were retained. The molecular and cellular mechanisms altered by cannabis use or THC/CBD exposure were then identified, including neural development and function, cytoskeletal function, cell adhesion, mitochondrial biology, inflammatory related pathways, lipid metabolism, the endocannabinoid system, the hypocretin/orexin system, and apoptosis. Alterations to those activities were also described in 19 of 228 focused on BDI.

**Conclusions:**

The biological mechanisms described in this study may be good candidates to the search for diagnostic biomarkers and therapeutic targets for BDI. Because cannabis use can trigger the onset of BD, further studies would be of interest to determine whether they are involved in the early development of the disorder, prompting early treatment.

## INTRODUCTION

Bipolar disorder type-I (BDI) is a severe psychiatric disorder involving cyclic mood oscillations (Shinozaki and Potash, 2014). If not properly treated, it can lead to suicide in 15% of cases (Andreazza and Young, 2014). This pathology lies in the leading positions for morbidity and for a loss of productivity according to the World Health Organization (Dusetzina et al., 2012). Genetics is an important risk factor; twin studies reported 85% of heritability (McGuffin et al., 2003; Kieseppä et al., 2004). Understanding how the identified susceptibility genes affect cell behavior and how they are influenced by the environment remains a challenge (Ferreira et al., 2008; Sklar et al., 2011; Craddock and Sklar, 2013).

Cannabis is a significant environmental risk factor in psychiatry (Hasin et al., 2016), particularly in the development of psychosis and mania (González-Pinto et al., 2008; Lagerberg et al., 2014). Individuals with BDI often experience psychotic symptoms and are at a higher risk of cannabis use (CU). Indeed, a recent study using artificial intelligence approaches to predict BD found that CU was among the most important predictors (Rabelo-da-Ponte et al., 2020). A similar situation arises with cannabis use disorder (CUD), indicating that BD and CUD share one of the highest comorbidities among all psychiatric disorders (Lalli et al., 2021). Indeed, 17% of people with BD have a lifetime CUD in contrast to 6% of the general population, and 7.2% of BD patients have had a CUD for the past year as opposed to 1.2% of the general population (Jordan Walter et al., 2021). Similarly, 11.5% of people with mania had a lifetime history of CU, and 6.9% were current cannabis users (Zorrilla et al., 2015).

A recent meta-analysis showed that CU was associated with a 3-fold increased risk of developing BD (Rodriguez et al., 2021). Furthermore, the impact of cannabis on BD is not only linked to the onset of the disorder but also to a worse clinical course. Thus, the assessment of how cannabis affects the outcome of BD has noted that symptoms remained more intense for a longer duration in cases with a history of CU (Strakowski et al., 2007). Nevertheless, although cannabis has been consistently reported as a risk factor for BD (Baethge et al., 2008; Richardson and Garavan, 2011), the biological mechanisms underlying this interaction remain unclear. Considering the shift toward the legalization of CU and the change in attitude regarding its safety and acceptability, this issue is important to resolve. Indeed, the rates of CU continue to rise worldwide (Hasin, 2018; Kraus et al., 2018), and a logical consequence would be an exacerbation of the impact on BD patients.

Accordingly, the goal of this study was to conduct 2 systematic reviews into the molecular and cellular alterations reported in cannabis users and in BDI patients, evaluating potential relationships between them. Thus, studying the common molecular/cellular alterations produced by cannabis and in BDI should help to identify related biological processes that might explain clinical psychopathology, guiding preventative strategies for BD patients who use cannabis.

## METHODS

### Search Strategy

This systematic review followed the PRISMA statement (Page et al., 2021) and involved a dual systematic search of the literature indexed in PubMed and Web of Science up until January 2023. We searched for articles that studied gene or protein expression in samples from chronic users of cannabis or from human cells after in vitro exposure to delta-9-tetrahydrocannabinol (THC) or cannabidiol (CBD). Moreover, a search for articles that studied human samples from patients with BDI was conducted.

The terms used to search for studies focused on cannabis were the following: (“cannabis” or “marijuana”) AND (“genetic expression” or “gene expression” or “proteomic” or “microarray” or “RNA-seq” or “transcription” or “transcriptomic” or “epigenomic”) AND (“olfactory neuroepithelium” or “iPSC” OR “induced pluripotent stem cells” or “lymphoblastoid cell” or “blood”) NOT (“mice” or “mouse” or “rat” or “rats”). For the second search, the following terms were used to identify potentially eligible publications related to BD: (“bipolar disorder”) AND (“genetic expression” or “gene expression” or “proteomic” or “microarray” or “RNA-seq” or “transcription” or “transcriptomic” or “epigenomic”) AND (“olfactory neuroepithelium” or “iPSC” OR “induced pluripotent stem cells” or “lymphoblastoid cell” or “blood”) NOT (“mice” or “mouse” or “rat” or “rats”).

Eligible for inclusion were only original articles published in English. Duplicates, reviews, and books were removed, and the title, abstract, and methods of the remaining studies were then screened. There were no restrictions on study setting or geographical location. Only studies that analyzed gene or protein expression were included for further review. In the case of articles investigating cannabis exposure, the additional inclusion criteria were studies on current cannabis users or studies in which the effects of cannabis on human cells in culture were tested. Additional exclusion criteria were studies on animal models or studies in which other substances were used. For articles investigating BDI, an additional inclusion criterion was that of studies investigating biological samples from individuals with BDI compared with controls. One author conducted the initial screening and data collection from the included articles, and a second author independently verified the accuracy of the described data during the revision process.

## RESULTS

### Search Results

**Figure 1.**
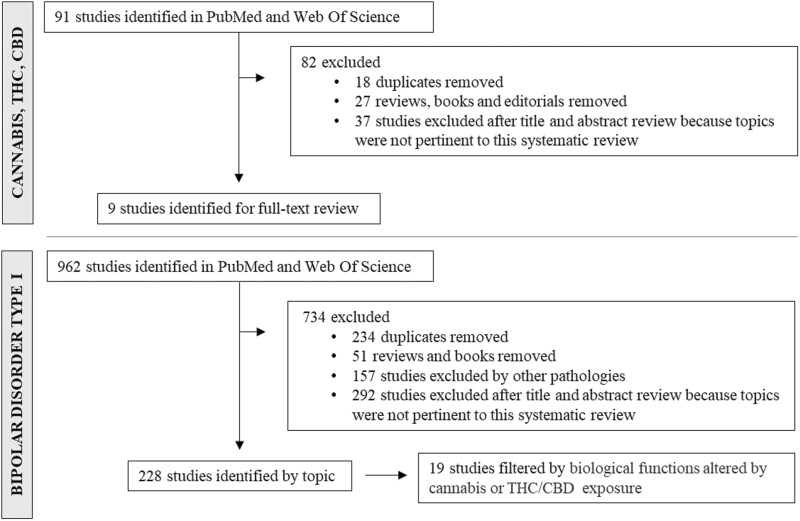
Flowchart of the selection procedure followed to identify relevant articles.

The initial search returned 91 reports on CU and 962 reports focused on BDI (Figure 1), from which duplicates and non-original articles were removed. Regarding CU, articles with data on smoked cannabis and studies that assessed treatment with cannabinoid derivatives in vitro were included, whereas studies addressing pathologies other than BDI were excluded from the BDI search. After reviewing the title and abstract, any article addressing topics other than those of interest were excluded. A total of 9 articles met all the inclusion criteria and focused on the molecular pathways or cellular changes induced by CU or THC/CBD exposure in vitro (Figure 1). We used the biological functions seen to be altered by cannabis or THC/CBD exposure as a filter (Table 1) to find articles in which these alterations were reported in BDI patients. From the 228 articles related to BDI, 19 described alterations to these mechanisms and were included (Figure 1). The information extracted from each article included the first author, year of publication, diagnosis, sample type, number of subjects, sex and average age, techniques for measurement, the biological mechanisms altered, and the main findings (Table 2).

**Table 1.** Molecular Pathways and Cellular Mechanisms Affected by Cannabis/THC/CBD Exposure

Molecular pathway or cellular mechanism	Related publications
1. Neural development and function: cell proliferation, synaptic function and cellular excitability.	Ghovanloo et al., 2018 Guennewig et al, 2018 He et al., 2019 Delgado-Sequera et al., 2021
2. Cytoskeletal function (cell morphology and microtubule dynamics)	Delgado-Sequera et al., 2021
3. Cell adhesion	Delgado-Sequera et al., 2021
4. Mitochondrial biology	Guennewig et al, 2018
5. Pathways related to inflammation and the production of nitric oxide (NO) or reactive oxygen species (ROS) by macrophages	Alasmari et al., 2021 Barrera-Conde et al., 2021
6. Lipid metabolism	Jayanthi et al., 2010 Alasmari et al., 2021
7. Endocannabinoid and hypocretin/orexin systems	Rotter et al., 2012 Rotter et al., 2013
8. Apoptosis	Delgado-Sequera et al., 2021

**Table 2.** Data Extraction From Studies Describing the Molecular and Cellular Changes Induced by Cannabis Use or in Vitro Exposure to THC or CBD (White) and in Bipolar Disorder Type I (Grey)

Author/y	Subject diagnosis	Sample type	Sample size (n)	Sex (m/f)	Average age (y)	Experimental techniques	Biological mechanisms	Main findings
1. Alasmari et al., 2021	CUD	Serum	C: 10 CUD: 10	C: 10/0 CUD: 10/0	C: 30 CUD: 25	WB, MS/MS analysis	Inflammatory related pathways and production of NO and ROS in macrophages Lipid metabolism	Altered proteins involved in immune system process, metabolic process and activation of (LXR/RXR) and (FRX/RXR)
2. Barrera-Conde et al., 2021	SCZ, CU SCZ/CU	ONEs	C:5 CU: 5 SCZ:5 SCZ/CU:5	C:3/5 CU: 4/1 SCZ:2/3 SCZ/CU:5/0	C:31 CU: 29 SCZ:37 SCZ/CU:41	Proteomic analysis	Inflammatory related pathways and production of nitric oxide (NO) and reactive oxygen species (ROS) in macrophages	Altered proteins involved in immunological processes
3. Chen at al., 2014	BDI	iPSCs	C:3 BD:3	NA	NA	IF, microarray, calcium signalling	Neural development and function: cellular proliferation, synaptic function, and cellular excitability Cytoskeleton function (cell morphology and microtubule dynamics) Cell adhesion	Differential expression in genes related to axon growth, synapse organization, calcium signaling, neurotransmitters, and their receptors. Differential transcripts involved in “cytoskeleton related processes,” “cell adhesion,” “cell-cell,” and “cell matrix interactions”
4. Delgado-Sequera et al., 2021	CU	ONEs	C:7 CB:6	C:5/2 CB:4/2	C:32 CB:29	IF, MS/MS analysis, WB, FC	Neural development and function: cellular proliferation, synaptic function, and cellular excitability Cytoskeleton function (cell morphology and microtubule dynamics) Cell adhesion Apoptosis	↓ Annexin V ↓ Iodine propidium ↑ Ki67 ↓ Vinculin ↑ Cell size
5. Escelsior et al., 2022	BD I, MDD	PBMCs	C: 25 BD:22 MDD: 22	C: 12/13 BD: 11/11 MDD: 10/12	C: 41 BD:46 MDD: 45	qRT-PCR	Endocannabinoid system	↓ CB1R
6. Fries et al., 2017a	BD I	LCLs	C: 20 BD: 22	C: 12/8 BD: 15/7	C: 35 BD: 34	qRT-PCR, Genome-wide expression	Mitochondrial DNA	↑ mtDNA copy number
7. Fries et al., 2017b	BD I	LCLs	C: 17 BD: 62	C: 10/7 BD: 24/38	C: 37 BD: 43	qRT-PCR, Genome-wide expression	Apoptosis	Altered 26 genes involved in apoptosis signaling: ↑ 7 ↓ 19
8. Ghovanloo et al., 2018	Control (treatment THC and CBD in vitro)	HEK-293 cells and iPSC	C: NA	C: NA	C: NA	Patch-clamp and IF	Neural development and function: cellular proliferation, synaptic function, and cellular excitability	CBD inhibits other voltage-dependent currents from diverse channels
9. Guennewig et al., 2018	SCZ (treatment THC in vitro)	iPSC	C:5 SCZ:4	C:NA SCZ:NA	C:NA SCZ:NA	RNA sequencing, quantitative RT–PCR	Neural development and function: cellular proliferation, synaptic function, and cellular excitability	↓ KCNE4, KCNA4 ↑ KCNJ10, KCNN3
10. He at., 2019	CU	Blood	C:90 CB:100	C:40/60 CB:73/17	C:23 CB:23	qPCR, eQTL, genome expression	Neural development and function: cellular proliferation, synaptic function, and cellular excitability	↑ PPFIA2
11. Jayanthi et al., 2010	Heavy marihuana user	Serum	C: 24 MJ: 18	C: 7/17 MJ: 10/8	C: 22 MJ:21	MS/MS analysis, IP	Lipid metabolism	↑ ApoC-III
12. Kathuria, el al., 2020	BD I	iPSHC	C:8 BD: 8	C: 6/2 BD: 5/3	18- 65	IHC, Transcriptome analysis	Neural development and function: cellular proliferation, synaptic function, and cellular excitability Cell adhesion	↓Response to stimulation and depolarization in BDI cerebral organoids ↓ NCAN
13. Li and El-Mallak et al.,, 2004	BD I	LCLs	C:15 BD:15	C: 5/10 BD: 5/10	BD: 33 C: 31	EF, WB	Neural development and function: cellular proliferation, synaptic function and cellular excitability	+ [Na] after ethacrynic acid stimulation
14. Madison et al., 2015	BD I	IPSCs	C: 2 BD:2	C: 1/1 BD: 0/2	BD: NA C: NA	Patch-clamp, Network analysis, clustering and gene ontology analysis	Neural development and function: cellular proliferation, synaptic function, and cellular excitability Cytoskeleton function (cell morphology and microtubule dynamics)	Altered CXCR4, CACNA1G, CACNA1E, CACNG8, CACNB1, SCN3A, SCN2A Alteration in “cell morphogenesis”
15. Matigian et al., 2007	BD I	LCLs	C:3 BD:3	C: ½ BD: ½	BD: 40 C: 40	Microarray, qRT-PCR	Apoptosis	Upregulation of “apoptosis process” ↑ Proapoptotic factors
16. McCurdy et al., 2006	BD I, SCZ	ONEs	C: 9 BD: 8 SCZ: 10	C: 5/4 BD: 4/4 SCZ: 7/3	C: 37 BD: 40 SCZ: 40	Microarray, IF	Apoptosis	↑ Death cells ↓ dUTP
17. Rotter et al., 2013	CU	PBMCs	C:21 CU:20 CS:36	C: 15/6 THC: 14/6 CS: 28/8	C: 31 THC: 33 CS: 32	PCR	Endocannabinoid system (CB1 and CB2 receptors expression and Orexin-A expression)	↑ CB1 mRNA expression levels and promoter methylation status
18. Rotter et al., 2012	CU	PBMCs	NS: 21 C: 20 CU: 36	NS: 15/6 C: 14/6 THC: 28/8	NS: 31 C: 33 THC: 32	PCR	Endocannabinoid system (CB1 and CB2 receptors expression and Orexin-A expression)	↓ Orexin A
19. Scaini et al., 2017	BD I	PBMCs	C: 16 BD: 16	C: 8/8 BD:8/8	C: 33 BD: 35	RT-PCR, Immunosorbent assay, quantification intrinsic apoptotic pathway Kit	Mitochondrial biology Apoptosis	↓ Opa1, Mfn2 ↑ Fis1 Negative correlation between mitochondrial fission/fusion proteins and apoptotic markers. Positive correlation between Mfn2 and Opa1 with mitochondrial content markers ↓ Bcl-xL/Bak, survivin, Bcl-xl, + Caspase-3
20. Scaini et al., 2019	BD I	PBMCs	C: 25 BD: 31	C: 7/18 BD: 7/24	C: 37 BD: 37	RT-PCR, Immunosorbent assay	Mitochondrial biology Apoptosis	↑ TSPO pathway proteins Mitochondrial dysfunction and related apoptosis
21. Song et al., 2015	Depressed BD I, manic BD I, Euthymic BD I	Plasma	D BD: 20 M BD: 15 E BD: 10 C: 20	D BD: 8/12 M BD: 6/9 E BD: 4/6 C: 8/12	D BD: 27 M BD: 29 E BD: 28 C: 28	MS/MS analysis, WB	Lipid metabolism	↓ Apo A1 ↑ ApoL1
22. Vadodaria et al., 2021	BD I	Plasma Astrocytes iPSCs derived	C:4 BD R:3 BD NR:3	C:4/0 BD R:3/0 BD NR:3/0	BD R:57-65 BD NR:22-69 C:NA	Sandwich immunoassay with V-PLEX Flow cytometry assay	Neural development and function: cellular proliferation, synaptic function, and cellular excitability synaptic function and cellular excitability Inflammatory-related pathways and production of NO and ROS in macrophages	↓ Activity in neurons co-cultured with nonstimulated and IL-1β–stimulated BD astrocytes. ↑ Number of IL-6–positive astrocytes after IL-1β and TNF-α stimulation. ↑ IL-6 levels ↑ IL-6 secretion under basal conditions and after stimulation with IL-1β
23. Voinsky et al., 2019	BD I	PBMCs	C:25 BD I:21	All female	C:NA BD I:39	RT-PCR	Neural development and function: cellular proliferation, synaptic function, and cellular excitability	↑ SCN11A
24. Washizuka et al., 2003	BD I, BDII	LCLs	C: 11 BD I: 13 BD II: 8	C: 8/3 BD I: 7/6 BD II: 2/6	C: 51 BD I: 52 BD II: 57	D-HPLC, RT- PCR	Mitochondrial biology	↓ mRNA expression NDUFV2
25. Washizuka et al., 2005	BD I, BDII	LCLs	C: 11 BD I: 13 BD II: 8	C: 8/3 BD I: 7/6 BD II: 2/6	C: 51 BD I: 51 BD II: 58	RT-PCR	Mitochondrial biology	↓ mRNA expression complex I subunit genes ↓ Expression levels; these genes correlated with NDUFV2
26. Washizuka et al., 2009	BD I, BD II, SCZ	LCLs	C: 33 BD I: 25 BD II: 10 SCZ: 13	C:22/11 BD I: 15/10 BD II: 2/8 SCZ: 9/4	C: 48 BD I: 52 BD II: 57 SCZ: 52	RT-PCR	Mitochondrial biology	No differences NDUFV2 mRNA levels in White BD
27. Wieck et al., 2016	BD I	PBMCs	C: 9 BD: 8	All females	C: 48 BD: 46	FC	Inflammatory-related pathways and production of NO and ROS in macrophages and apoptosis Inflammatory-related pathways and production of NO and ROS in macrophages	↑ MAPK, p-ERK, p-NF KB, ↑ IL-12, p70 ↑ TNF-α ↓ IL-6
28. Zain et al., 2012	BD I	PBLs	C: 16 BD:16	C: 8/8 BD:6/10	C: 44 BD:40	PCR	Neural development and function: cellular proliferation, synaptic function and cellular excitability	↓ PDLIM5 mRNA expression

Abbreviations: BDI, bipolar disorder type I; BDII, bipolar disorder type II; C, control; CU, cannabis users; CUD, cannabis use disorder; D-HPLC, denaturing high performance liquid chromatography; FC, flow cytometry; IF, immunofluorescence; IP, immunorecipitation; LCLs, lymphoblastoid cell lines; MDD, major depressive disorder; MS/MS, tandem mass spectrometry; NA, not available; NO, nitric oxide; ONEs, olfactory neuroepithelium cells; PBMCs, peripheral blood mononuclear cells; qPCR, quantitative polymerase chain reaction; ROS, reactive oxygen species; RT-PCR, real time polymerase chain reaction; SCZ, schizophrenia; WB, western blot.

### Neural Development and Function: Cell Proliferation, Synaptic Function, and Cellular Excitability

#### Cannabis

Alterations to neural development and functioning as a consequence of exposure to cannabis or THC/CBD were described in 4 articles. A proteomic analysis of the olfactory neuroepithelium, a tissue that contains neural precursors that can be cultured, identified the differential expression of proteins related to cell proliferation in cannabis users compared with controls. After performing immunofluorescence studies, they observed that the neural precursors of cannabis users showed an increase in the proliferation (Delgado-Sequera et al., 2021).

An enhanced expression of liprin-α-2 mRNA (*PPFIA2*) in blood samples from heavy cannabis users was also found (He et al., 2019). *PPFIA2* encodes liprin-α-2, a protein involved in the growth of excitatory synapses and establishment of dendritic spines. Interestingly, *PPFIA2* levels were positively associated with cannabis consumption and negatively correlated with estimated intelligence (He et al., 2019).

The effect of CBD on human-induced pluripotent stem cells (iPSCs) and HEK-293 cells was also explored electrophysiologically (Ghovanloo et al., 2018). CBD blocked sodium and potassium currents mediated by hNav1.1-1.7, hNav1.2, and Kv2.1, which could reflect changes in cell gating provoked by altering membrane fluidity or alterations to direct interactions with sodium and potassium channels (Ghovanloo et al., 2018). Another study analyzed the effect of THC on iPSCs using RNA-sequencing (RNA-seq) to identify changes in gene expression. Network analysis of all the differentially expressed genes (DEGs) after THC incubation showed changes in pathways involved in synaptic function (Guennewig et al., 2018). Specifically, acute THC exposure provoked changes to genes encoding potassium voltage-gated channels (*KCNE4*, *KCNA4*, *KCNJ10*, and *KCNN3*) and genes that influence the postsynaptic density (*HOMER1*, *GRID2*, *GRIK1*, and *SIPA1L1*), whereas chronic THC exposure affected other ion channels (*KCNJ2*, *KCNA2*, and *KCNT2*) and additional synapse-related genes (*SYNGAP1* and *SHANK1*).

#### Bipolar Disorder

Altered neural development and activity was described in 8 papers on BDI patients, one of which examined differences in the transcriptome of neurons derived from neuronal rosettes obtained from BDI patients and controls (Chen et al., 2014). DEGs in cells derived from BDI patients were related to neural differentiation, axon growth, synapse organization, calcium signaling, neurotransmitters, and their receptors (Chen et al., 2014). A family-based paradigm was applied elsewhere to characterize iPSCs from 2 BDI-affected brothers and their 2 unaffected parents (Madison et al., 2015). When neural progenitor cells (NPCs) were differentiated into neurons, those derived from BDI patients displayed a specific deficit in their proliferation and differentiation, whereas non-neural linages were not affected. Accordingly, DEGs related to neurodevelopment were evident when iPSCs, NPCs, and their derived neurons were analyzed (Madison et al., 2015). In contrast to the deficient proliferation of NPCs derived from BDI patients observed in 2 studies (Madison et al., 2015; Song et al., 2015), no such difference in proliferation (mitotic state) or related proteins was observed in olfactory neuroepithelial cells (ONEs) derived from BDI patients relative to controls (McCurdy et al., 2006).

Several DEGs were identified in BDI patient iPSC-derived neurons encoding the *SCN2A* and *SCN3A* sodium channels and calcium channels or ion channel subunits, such as *CACNA1G*, *CACNA1E*, *CACNB1*, and a regulator of AMPA receptor localization (Madison et al., 2015). A shift in *CACNA1E* expression was observed in both these studies, albeit decreasing in 1 study (Madison et al., 2015) whereas increasing in the other (Chen et al., 2014). In addition, expression of the *FGF14* gene was enhanced in neurons from BDI patients (Chen et al., 2014; Madison et al., 2015), with *FGF14* regulating the activity of presynaptic calcium channels. Hence, calcium signaling may be altered in neurons derived from BDI patients, and, moreover, the CACNA1G and CACNA1E calcium channel subunits may be key mediators.

Differences in sodium channels or currents have been found elsewhere in cells derived from BDI patients (Li and El-Mallakh, 2004; Voinsky et al., 2019). Abnormalities in the expression and activity of the sodium pump were studied in lymphoblastoid cell lines (LCLs) derived from BDI patients, specifically in the presence of ethacrynic acid (Li and El-Mallakh, 2004). In response to ethacrynic acid, the expression and activity of the sodium pump increased in the control cells to regulate their intracellular Na^+^ concentration. However, neither the expression nor the activity of the sodium pump changed in response to ethacrynic acid in cells from BDI patients, resulting in an increase in intracellular Na^+^. Thus, BDI might be related to a failure to regulate sodium homeostasis in the cells (Li and El-Mallakh, 2004). Moreover, stronger *SCN11A* expression (NaV1.9 voltage-gated sodium channel) was detected in both peripheral blood mononuclear cells (PBMCs) and gene databases of postmortem brains from BDI patients compared with controls (Voinsky et al., 2019). Hence, the NaV1.9 channel could possibly serve as a biomarker for this pathology.

In addition, weaker *PDLIM5* expression was found in peripheral blood leukocytes (PBLs) derived from BDI patients than in controls. *PDLIM5* encodes the LIM domain protein that promotes dendritic spine shrinkage, and it has previously been related to the pathophysiology of BDI (Zain et al., 2012). Elsewhere, an RNA-seq analysis of cerebral organoids derived from BDI patients and controls demonstrated significant differences in genes modulating the inhibitory/excitatory balance in the brain, as well as down-regulation of pathways involved in processes like “synaptic biology” and “neuronal development” (Kathuria et al., 2020). The electrical activity of cerebral organoids derived from BDI patients diminished in response to electrical stimulation and depolarization relative to controls (Kathuria et al., 2020). Furthermore, iPSCs-derived astrocytes from BDI patients affected the electrical activity of neurons, dampening the activity of a neuronal cell line in co-cultures (Vadodaria et al., 2021).

### Cytoskeletal Function (Cell Morphology and Microtubule Dynamics)

#### Cannabis

A proteomics analysis of ONEs from cannabis users showed that “microtubule dynamics,” “actin cytoskeleton signaling,” and “cell growth” were altered relative to control. In addition, the cells of cannabis users were larger than those of control, possibly due to cytoskeletal changes (Delgado-Sequera et al., 2021).

#### Bipolar Disorder

Three articles detected alterations related to the cytoskeleton in cells derived from BDI patients. Transcripts involved in “cell morphogenesis” related to neuronal differentiation and “cytoskeleton-related processes” were identified in iPSC-derived neural progenitors and iPSC-derived neurons from BDI patients (Chen et al., 2014; Madison et al., 2015). Moreover, *PDLIM5*, a protein related to actin reorganization and involved in the morphogenesis of dendrites, was expressed more weakly in PBLs derived from BDI patients (Zain et al., 2012).

### Cell Adhesion

#### Cannabis

In a proteomic analysis of ONEs from cannabis users, “cell adhesion capacity” was one of the pathways altered relative to the controls (Delgado-Sequera et al., 2021). In addition, immunofluorescence studies indicated a decrease in the focal adhesion (vinculin^+^) to the substrate of ONEs from cannabis users.

#### Bipolar Disorder

Altered cell adhesion was described in 3 articles from BDI, the latest a transcriptomic study in which RNA-seq analysis showed a downregulation of pathways involved in cell adhesion in BDI iPSC-derived cerebral organoids (Kathuria et al., 2020). Subsequent analysis of DEGs showed that *neurocan* (*NCAN*), a proteoglycan of the neuronal extracellular matrix that modulates migration and neural adhesion, was significantly downregulated in organoids from BDI patients (Kathuria et al., 2020). In other studies, iPSCs derived from individuals with BDI showed that DEGs were associated with “cell adhesion,” “cell–cell,” or “cell–matrix interactions” (Chen et al., 2014; Madison et al., 2015).

### Mitochondrial Biology

#### Cannabis

Transcriptomic analysis of iPSCs-derived neurons exposed to THC highlighted DEGs related to “mitochondrial biology,” with the specific upregulation of mitochondrial mRNAs following acute (*MT-CO1* and *MT-CO3*) or chronic (*COX7A2*) exposure to THC (Guennewig et al., 2018).

#### Bipolar Disorder

Six articles studied alterations to “mitochondrial biology” in BDI. An association between a polymorphism in the nuclear-encoded mitochondrial complex subunit-I gene *NDUFV2* and BDI was first established in a Japanese population, suggesting that it could be a pathophysiological risk factor (Washizuka et al., 2003). Complex-I is a component of the electron transport chain, and it fulfils a central role in free radical biology and cellular bioenergetics. In addition, less expression of *NDUFV2* and other complex-I subunit genes in LCLs from BDI patients was found, although no such differences were observed in a White population (Washizuka et al., 2003, 2005, 2009).

Two other studies focused on the relationship between the changes in mitochondrial dynamics and early apoptotic events (Scaini et al., 2017, 2019), highlighting the downregulation of the mitochondrial fusion-related proteins OPA1 and MFN2 in PBMCs from BDI patients and the upregulation of the fission protein Fis1. In addition, a significant decrease in citrate synthase activity was found. A negative correlation between mitochondrial fission/fusion proteins and apoptotic markers was described, as well as a positive correlation between markers of mitochondrial content and MFN2 or OPA1 (Scaini et al., 2017). Changes in the 18kDa translocator protein–related pathway were also assessed, playing an important role in regulating mitochondrial function (Scaini et al., 2019).

Finally, BDI patients had higher mitochondrial DNA (mtDNA) copy numbers, which significantly correlated with epigenetic age acceleration in older patients, suggesting that BDI may be associated with accelerated aging (Fries et al., 2017a).

### Pathways Related to Inflammation and Production of Nitric Oxide (NO) or Reactive Oxygen Species (ROS) by Macrophages

#### Cannabis

A proteomic analysis performed on both neural progenitors and serum from cannabis users described an effect on the “immune system,” specifically proteins related to the interleukin-12 complex and enriched canonical pathways: “activation of liver X receptors/retinoid X receptor” (LXR/RXR), “farnesoid X receptors/retinoid X receptor activation” (FRX/RXR), “acute phase response signaling,” “atherosclerosis signaling and production of NO,” and “ROS in macrophages” (Alasmari et al., 2021; Barrera-Conde et al., 2021).

#### Bipolar Disorder

Two articles reported the modulation of the immune system in BDI patients, in 1 of which the iPSC-derived astrocytes from BDI patients or controls were used to assess the inflammation-related phenotypes (Vadodaria et al., 2021). Neuron activity diminished when they were co-cultured with astrocytes derived from BDI patients. Under basal conditions, more IL-6 was secreted by BDI astrocytes, while the IL-1β-induced genes were common to both BDI and controls. Following IL-1β and TNF-α stimulation, more IL-6–positive astrocytes were derived from BDI patients. Hence, there appears to be a role for astrocytes in the neuroinflammation associated with psychiatric disorders, which is relevant to the altered IL-6 and inflammatory signaling in BDI patient astrocytes (Vadodaria et al., 2021).

In addition, the frequencies of toll-like receptors (TLR) were analysed, a family of receptors constituting the first line of defense against microbes. Higher proportions of TLR-1^+^ and TLR-2^+^ monocytes were detected in BDI patients in conjunction with reduced TLR-5. Moreover, levels of IL-8, IL-12p70 and TNF increased after stimulation with TLR-1, TLR-2, and TLR-6 agonists, suggesting enhanced signaling via these receptors in BDI. The proportion of TLR-2^+^ Treg cells and activated T-cells expressing both TLR-2 and TLR-5 also increased in BDI patients, suggesting TLR receptors are involved in the inflammatory processes described in BDI (Wieck et al., 2016).

### Lipid Metabolism

#### Cannabis

Alterations related to lipid metabolism were detected after a proteomic analysis of serum samples from BDI patients and controls, indicating that “acute phase response signaling” and “atherosclerosis signaling” were related to metabolic changes (Alasmari et al., 2021). The most interesting enriched canonical pathways included LXR/RXR activation, which is involved in cholesterol metabolism, whereas FRX/RXR activation is mainly involved in the metabolism of lipids and glucose (Alasmari et al., 2021). In a proteomic analysis of serum samples from cannabis users, all 3 isoforms of ApolipoproteinC-III (ApoC-III) were enhanced in cannabis users relative to controls. ApoC-III regulates the catabolism of lipoproteins rich in triglycerides, and increased levels of this protein was proposed as a risk factor for cardiovascular disorders (Jayanthi et al., 2010).

#### Bipolar Disorder

One article studied alterations to lipid metabolism in BD, performing a proteomic analysis on plasma samples in various BDI mood states [depressed, manic, and euthymic (Song et al., 2015)]. Of the 32 proteins identified, 16 were altered in BDI relative to the controls independent of the mood state, whereas the rest of the proteins were specifically associated with a particular BDI mood state. Lower Apo-A1 and higher Apo-L1 levels were detected in the plasma from BDI patients relative to the control, whereas CA-1 was downregulated only in depressed BD patients. ApoA1 is the main structural component of high-density lipoproteins, and ApoL is found in high-density lipoprotein complexes that play a central role in cholesterol transport. Conversely, CA-1 is a carbonic anhydrase isoenzyme that catalyzes the CO_2_/HCO_3_ conversion. Therefore, it was proposed that BDI pathophysiology may be associated with early alterations to lipid metabolism irrespective of the mood state, whereas CA-1 might be involved in the depressive episodes (Song et al., 2015).

### Endocannabinoid System and Hypocretin/Orexin System

#### Cannabis

Considering that the relationship between cannabinoid receptors and neuropeptides-like Orexins that are involved in feeding regulation, *Orexin-A* mRNA expression and promoter methylation was measured in cannabis users and controls. *Orexin-A* expression was downregulated in PBMCs from cannabis users relative to the controls, although *Orexin-A* promoter methylation did not differ (Rotter et al., 2012). The consequences of cannabis dependence on endocannabinoid receptors was studied, evaluating *CB1* and *CB2 receptor* (*CB1/2R*) expression and promoter methylation in the PBMCs from cannabis users and controls [nonsmokers and tobacco smokers (Rotter et al., 2013)]. *CB1R* mRNA levels were lower in cannabis users than in controls, and its promoter was more heavily methylated (Rotter et al., 2013). In addition, a negative correlation was found between the levels of *CB1R* methylation and its mRNA expression such that more promoter methylation was associated with weaker *CB1R* mRNA expression. No such changes were observed for the *CB2R* (Rotter et al., 2013).

#### Bipolar Disorder

*CB1R* expression has been assessed in BDI, evaluating its expression in PBMCs from patients classified between mania and depression states (Escelsior et al., 2022). *CB1R* expression was stronger and less variable in the manic state than in controls, whereas *CB1R* expression was weaker and more variable in the depressive state.

### Apoptosis

#### Cannabis

The proteomic analysis performed in NPCs from the olfactory neuroepithelium showed differential expression of proteins related to “apoptosis” in samples from cannabis users. Fewer apoptotic and necrotic cells in cannabis users compared with controls were quantified by flow cytometry (Delgado-Sequera et al., 2021).

#### Bipolar Disorder

Five articles reported alterations in apoptosis in samples from BDI patients. Gene expression profiling was performed to identify genes dysregulated in LCLs or PBMCs from BDI patients. An increase in pro-apoptotic genes and a decrease in anti-apoptotic proteins were described in BDI patients compared with control (Matigian et al., 2007; Scaini et al., 2017, 2019). These findings are consistent with results from ONEs, where more dying cells were evident based on their nuclear staining (McCurdy et al., 2006).

Finally, 1 study examined the effects of lithium-induced gene expression in BDI patients’ LCLs (Fries et al., 2017b). Exposure of LCLs to lithium altered the expression of 236 genes, highlighting an enrichment of genes related to cell death and programmed cell death regulation in patients’ LCLs. Hence, lithium appears to modulate apoptosis specifically in BDI but not in LCLs from controls (Fries et al., 2017b).

## DISCUSSION

The objective of this study was to explore the biological mechanisms triggered by CU that may contribute to the vulnerability of BDI, with the goal of identifying potential biomarkers for improved diagnosis and early intervention. Our investigation described several molecular and cellular modifications induced by cannabis, encompassing neural function, cytoskeleton, cell adhesion, mitochondrial biology, inflammation, lipid metabolism, endocannabinoid, and hypocretin/orexin systems, as well as apoptosis. Subsequently, our examination unveiled several shared alterations in BDI patients. Following, we discuss these potential shared pathways affected by both CU and in individuals with BDI.

### Neural Function, Cytoskeleton, and Cell Adhesion

Exposure to cannabis or THC/CBD altered aspects of neuronal development and activity, enhancing the proliferation of neural precursors, perhaps by affecting the levels of proliferation-related proteins and the expression of genes related to synaptic function (Ghovanloo et al., 2018; Guennewig et al., 2018; He et al., 2019; Delgado-Sequera et al., 2021). Furthermore, CBD exposure blocks sodium and potassium currents in iPSCs (Ghovanloo et al., 2018). All these cellular mechanisms are important for neuronal function, and related alterations were found also in cells derived from BDI patients. iPSC-derived neuronal precursors from BDI patients showed deficient proliferation and weaker neuronal differentiation, and DEGs related to neural differentiation were identified (Chen et al., 2014; Madison et al., 2015). Interestingly, normal proliferation and differentiation was observed when iPSCs from BDI patients differentiated into a non-neuronal cell lineage, indicating a specific deficit in neuronal differentiation (Madison et al., 2015). This specific behavior of iPSC-derived neurons is consistent with the normal proliferation of ONEs derived from BDI patients, although it should be noted that such cells proliferate more when they are derived from schizophrenia patients or cannabis users (McCurdy et al., 2006; Delgado-Sequera et al., 2021). The regulation of other genes related to neuronal function was also altered in samples derived from BDI patients, such as genes involved in axon growth, synapse organization, dendritic spine shrinkage, calcium signaling, sodium/potassium channels, neurotransmitter release, and their receptors (Zain et al., 2012; Chen et al., 2014; Madison et al., 2015; Voinsky et al., 2019; Kathuria et al., 2020). In addition, electrophysiological and functional analysis of BDI-derived neurons revealed alterations in the activity of sodium pumps, a dampened response to electrical stimulation, and weaker neuronal activity (Li and El-Mallakh, 2004; Kathuria et al., 2020; Vadodaria et al., 2021). Together, these data suggest that cell excitability and synaptic function may be common cellular mechanisms altered by both BDI and CU.

Correct neural development and activity requires proper cytoskeletal function and cell adhesion (Leshchyns’Ka and Sytnyk, 2016; Lasser et al., 2018). The regulation of proteins related to the cytoskeleton and adhesion was altered in ONEs derived from cannabis users, with these cells showing altered morphology and diminished adhesive capacity, possibly related to cytoskeletal defects (Delgado-Sequera et al., 2021). Similarly, RNA-seq analysis of iPSC-derived neural precursors and neurons from BDI patients revealed DEGs related to the cytoskeleton, morphogenesis, and adhesion (Chen et al., 2014; Madison et al., 2015; Kathuria et al., 2020). In the case of brain organoids from iPSCs, cell adhesion pathways were downregulated, as was the expression of *NCAN* that modulates migration and neuronal adhesion (Kathuria et al., 2020). Other studies described alterations to the cytoskeleton and in adhesion in BD patient’s cells, such as altered microtubule organization, short microtubules, and weaker adhesion capacity of the ONEs derived from these patients (Solís-Chagoyán et al., 2013; Muñoz-Estrada et al., 2015). Taken together, these reports indicate that the cytoskeleton and cell adhesion are altered in both BD patients and cannabis users, although further analysis would be needed to establish a common relationship. Apart from these studies, a significant difference in *PDLIM5* expression was detected in PBLs from BDI patients (Zain et al., 2012). *PDLIM5* reduces dendritic spine head size in the postsynaptic density through a mechanism involving the actin cytoskeleton, and silencing this gene increases spine diameter. Indeed, it was suggested that alterations to *PDLIM5* may contribute to psychiatric disorders (Herrick et al., 2009). In terms of dendritic spine density, it is interesting to note that an increase in liprin-α2 has been found in blood samples from cannabis users (He et al., 2019). When this protein is depleted, it was seen to be involved in the organization of the synaptic scaffold at the presynaptic terminal (Spangler et al., 2013). Further studies will be needed to analyze whether an increase in this protein is observed in the neurons of cannabis users and its consequences in synapses, which could influence BD symptoms.

### Mitochondrial Biology

RNA-seq analysis of iPSC-derived neurons exposed to THC revealed DEGs related to “mitochondrial biology” (Guennewig et al., 2018). Indeed, THC enhances oxidative stress and mitochondrial dysfunction in the brain, which appears to be a risk factor for ischemic stroke (Wolff et al., 2014). Several lines of evidence suggest that brain energy metabolism, mitochondrial function, and redox balance are affected in psychiatric disorders (Kim et al., 2019). Pathways underlying neuropathology in BD include the dopaminergic system, inflammatory cytokines, oxidative and nitrosative stress, and mitochondrial dysfunction (Sigitova et al., 2017). In blood samples from BDI patients, the expression of genes related to mitochondrial function is altered, such as genes included in the Complex-I respiratory chain, mitochondrial fusion/fission, and mitophagy (Washizuka et al., 2003, 2005, 2009; Fries et al., 2017a; Scaini et al., 2019). In addition, a correlation between higher mtDNA copy number and epigenetic age acceleration was described (Fries et al., 2017a). Aging is associated with a progressive alteration in mitochondrial respiratory chain activity, and thus mtDNA quantification could represent an interesting biomarker for BDI (Fries et al., 2017a). Hence, although specific alterations to genes related to mitochondria are not common to THC exposure and BD, they do converge on producing mitochondrial dysfunction.

### Pathways Related to Inflammation

Inflammation is another mechanism altered by CU that is also affected in BDI patients. In this sense, proteomic studies conducted on cannabis users showed alterations in immune system molecules and signaling pathways (Alasmari et al., 2021; Barrera-Conde et al., 2021). Exogenous cannabinoids derived from *Cannabis sativa*, like THC or cannabigerol, have been shown to exert immunosuppression, providing evidence of its neuroprotective effects (Suárez-Pinilla et al., 2014; Gugliandolo et al., 2018).

As previously mentioned, increase in IL-6 in BDI patients may influence the dampened neural activity in co-cultures with astrocytes, and thus astrocytes may influence neuroinflammation in psychiatric disorders (Vadodaria et al., 2021). This concept is consistent with enhanced cytokine production and lymphocyte activation when PBMCs from BD patients are compared with those from controls (Do Prado et al., 2013). Additionally, the expression of several toll-like receptors was altered in BD patients, along with enhanced signaling via these receptors, suggesting their involvement in the inflammatory processes associated with this condition (Wieck et al., 2016). Although several studies have focused on the role of inflammatory-related pathways in BD, this appears to be the only study addressing the role of TLRs in BD.

Taken together, these data highlight that inflammation-related pathways are altered in both cannabis users and BDI patients, although we found alteration in the production of NO and ROS only in cannabis users.

### Metabolism of Lipids

Proteomic analysis on the serum from cannabis users described alterations to proteins and pathways involved in lipid metabolism (Jayanthi et al., 2010; Alasmari et al., 2021). Specifically, increased ApoA-I was evident in cannabis users (Jayanthi et al., 2010). Interestingly, changes in ApoA-I expression were also found in blood from BDI patients, although in the opposite direction to cannabis users (Song et al., 2015). Importantly, lithium enhanced the expression of this protein, and indeed it has been proposed as a biomarker of response to lithium (Sussulini et al., 2011). In addition, gender may also influence the levels of ApoA-I, which is higher in women than in men (Haenisch et al., 2014). Therefore, lipid metabolism seems to be affected in both cannabis users and BDI patients; considering that it has been proposed as a therapeutic target for lithium, further analysis will be of interest to identify biomarkers of BD risk and treatments that are specific to lipid metabolism.

### Endocannabinoid and Hypocretin/Orexin Systems

CU was associated with increased methylation of the CB1R promoter and decreased mRNA encoding this receptor (Rotter et al., 2013). Among BDI patients, *CB1R* gene expression was stronger and less variable in the manic patients than in the depressed group (Escelsior et al., 2022). These data may suggest that the depressive state in BDI follows a similar trend in terms of *CB1R* mRNA expression as in cannabis users. The expression of *CB1R* has been studied in major depressive disorder with mixed results, ranging from an increase in the prefrontal cortex to decreased levels or no differences in the anterior cerebral cortex (Navarrete et al., 2020). Moreover, some polymorphisms in the *CB1R* gene have been proposed as susceptibility factors for developing mood disorders (Navarrete et al., 2020). There are also conflicting results when trying to associate *CBR1* polymorphisms with the pathophysiology of BD, with some but not all studies identifying associations (Navarrete et al., 2020). However, the limited number of studies and these inconsistencies indicate a need for further analyses to better understand the relationship between the endocannabinoid system and BD.

A decrease in *Orexin-A* has also been associated with CU (Rotter et al., 2012), a protein related to the sleep-wake cycle. There is a well-established relationship between circadian cycle disturbance and BD, with many studies documenting sleep disturbances and circadian rhythm dysfunction in association with BD (Takaesu, 2018; Faltraco et al., 2021). Because circadian rhythm dysfunction is more prominent in BD than in major depressive disorder, circadian rhythm dysfunction is a characteristic marker of BD (Takaesu, 2018).

### Apoptosis

CU appears to decrease apoptosis (Delgado-Sequera et al., 2021), and BDI patients appear to have fewer anti-apoptotic and more pro-apoptotic molecules (Matigian et al., 2007; Scaini et al., 2019). It was suggested that dysregulated apoptosis may promote neuronal death and that this might underlie the pathophysiology of BDI (Matigian et al., 2007), with changes in the balance between active inhibitors of apoptosis and caspases driving altering the apoptotic programme (Scaini et al., 2019). Indeed, more dead cells were quantified among the cells derived from BDI patients than controls (McCurdy et al., 2006), and enhanced apoptosis was described elsewhere in BD patients (Uribe and Wix, 2012; Pietruczuk et al., 2018). Furthermore, apoptosis was proposed as a therapeutic target for lithium (Fries et al., 2017b). Although the molecular mechanisms affected by lithium are not fully understood, there are data supporting an anti-apoptotic effect (Mishra et al., 2023).

Both THC and CBD reduced cell proliferation and induced apoptosis in cancer cells (Pellati et al., 2018; Hosami et al., 2021; Lal et al., 2021; Cherkasova et al., 2022) while protecting healthy tissue from such cell death (Bogdanović et al., 2017). Thus, cannabis and the clinical response to lithium may be associated with an anti-apoptotic effect (Bogdanović et al., 2017; Delgado-Sequera et al., 2021; Mishra et al., 2023). Further studies of CU in BD patients should focus on the effects on apoptosis and the possible therapeutic benefits.

### Limitations

We found some limitations to study the biological mechanisms affected in the brain by CU or BDI. First, many studies used blood samples that may not reflect brain function. Second, a substantial number of studies had a small sample size. Therefore, additional research with larger sample sizes should be performed. Third, this study did not assess the risk of bias in the selected articles, which may affect the robustness of the results. Last, studies in BD patients who also use cannabis would be of interest, but most usually focus on cognitive performance (Sagar et al., 2016; Jordan Walter et al., 2021). Thus, we still lack the biological information that would be useful to find biomarkers of BD risk or therapeutic targets, because CU increases the risk of both the onset and worsening of BD symptoms. Despite these limitations and in the absence of further specific studies, the strength of this review lies in providing a pioneering comparative description. The general findings of this review open new avenues for further in-deep research.

## CONCLUSIONS

The common mechanisms altered because of cannabis, THC, or CBD exposure and in BDI patients were related to cellular excitability and synaptic function, cytoskeleton and cell adhesion, mitochondrial dysfunction, inflammation, lipid metabolism, endocannabinoid system, and apoptosis. It would be very interesting to determine whether the alterations to these processes provoked by CU trigger a higher risk of BD in susceptible individuals and whether this susceptibility is due to alterations in these events. If so, they could be considered as early diagnostic biomarkers or therapeutic targets that would help in early treatment at the onset of BD.

## Data Availability

All the data extracted from included original articles are available in PubMed or Web of Science. This review was conducted without previous registration, and no protocol document was prepared.

## Interest Statement

Gonzalez-Pinto is a consultant or offered lectures from Janssen-Cilag, Lundbeck, Otsuka, Pfizer, Takeda, and Angelini.
